# Vitamin D Intake and Status in 6-Year-Old Icelandic Children Followed up from Infancy

**DOI:** 10.3390/nu8020075

**Published:** 2016-02-04

**Authors:** Birna Thorisdottir, Ingibjorg Gunnarsdottir, Laufey Steingrimsdottir, Gestur I. Palsson, Bryndis E. Birgisdottir, Inga Thorsdottir

**Affiliations:** 1 Unit for Nutrition Research, Faculty of Food Science and Nutrition, School of Health Sciences, University of Iceland and Landspitali University Hospital, Reykjavik 101, Iceland; ingigun@hi.is (I.G.); laufey@hi.is (L.S.); beb@hi.is (B.E.B.); ingathor@hi.is (I.T.); 2 Children’s Hospital, Landspitali University Hospital, Reykjavik 101, Iceland; gesturip@landspitali.is

**Keywords:** 25-hydroxyvitamin D, child, dietary supplements, seasons, tracking, vitamin D

## Abstract

High serum 25-hydroxyvitamin D (25(OH)D) levels have been observed in infants in Nordic countries, likely due to vitamin D supplement use. Internationally, little is known about tracking vitamin D status from infancy to childhood. Following up 1-year-old infants in our national longitudinal cohort, our aims were to study vitamin D intake and status in healthy 6-year-old Icelandic children (*n* = 139) and to track vitamin D status from one year of age. At six years, the mean 25(OH)D level was 56.5 nmol/L (SD 17.9) and 64% of children were vitamin D sufficient (25(OH)D ≥ 50 nmol/L). A logistic regression model adjusted for gender and breastfeeding showed that higher total vitamin D intake (Odds ratio (OR) = 1.27, 95% confidence interval (CI) = 1.08–1.49), blood samples collected in summer (OR = 8.88, 95% CI = 1.83–43.23) or autumn (OR = 5.64, 95% CI = 1.16–27.32) compared to winter/spring, and 25(OH)D at age one (OR = 1.02, 95% CI = 1.002–1.04) were independently associated with vitamin D sufficiency at age six. The correlation between 25(OH)D at age one and six was 0.34 (*p* = 0.003). Our findings suggest that vitamin D status in infancy, current vitamin D intake and season are predictors of vitamin D status in early school age children. Our finding of vitamin D status tracking from infancy to childhood provides motivation for further studies on tracking and its clinical significance.

## 1. Introduction

Vitamin D sufficiency in childhood is associated with improved bone health [1] and may possibly lower the risk for type 1 diabetes [2], cardiovascular disease [3] and other diseases later in life [4]. It may also have short-term benefits beyond bone metabolism. Some, but not all, studies have found cross-sectional associations between vitamin D status and lipid profile or other cardiometabolic risk factors in children [5,6,7]. Serum 25-hydroxyvitamin D (25(OH)D) is generally considered the best measure of vitamin D status and is believed to reflect the combined contributions from cutaneous vitamin D synthesis and vitamin D intake from the diet, *i.e.*, food naturally rich in vitamin D, fortified food and supplements [8,9].

Iceland has a small and relatively genetically homogenous population [10]. It is geographically located at 63–66°N, resulting in little or no cutaneous vitamin D synthesis during large parts of the year [11,12]. Similar to other northern regions, e.g., Norway, Sweden, Denmark, Finland [13], the USA [8,14] and Canada [15], the recommended vitamin D intake for children aged 2 weeks to 10 years is 10 µg/day. Daily use of cod liver oil or another form of vitamin D supplement is part of the Icelandic food based recommendations for all age groups [16].

In our previous publication from a cohort designed to examine diet, growth and health in infancy and childhood we reported high serum 25(OH)D levels among 1 year old infants [17]. We suggested that these high levels might be explained, at least in part, by regular use of vitamin D supplements and/or vitamin D fortified foods or drinks by 75% of the infants. A school-based lifestyle study among 7 year old Icelandic children reported lower vitamin D intake and also lower levels of 25(OH)D than among the infants [18]. This is in line with reports of lower adherence to nutritional guidelines and decreased use of supplements as children grow older [19,20,21]. Also, several cross-sectional epidemiological studies have reported lower 25(OH)D levels among older children and adolescents than infants and younger children [22,23,24]. It is unknown to what extent the apparent decrease in 25(OH)D with age can be explained by background characteristics due to different study groups, decreased vitamin D intake, or other factors. Studies are lacking that measure both vitamin D intake and status at more than one time-point in the same individuals, thus giving the possibility to study maintenance or tracking of vitamin D status from younger age to childhood. Following up with infants from our population-based longitudinal infant and child cohort [17], our aims were to study vitamin D intake and status in healthy 6 year old children and to track vitamin D status from 1 year of age.

## 2. Materials and Methods

### 2.1. Study Design and Subjects

The study population, recruitment and data collection have previously been described in detail [25,26]. In brief, a random sample of 250 Icelandic infants born in 2005 (6% of live born infants) was collected and followed throughout the first year of life. At 6 years, 172 participated in a follow-up. In this current analysis, eligible subjects were those with vitamin D status measured at 6 years, in total 139 children (81% of children in the follow-up). Data on vitamin D status at both 1 and 6 years of age were available for 74 children. The basic characteristics of eligible subjects did not differ from other subjects in the follow-up. Informed written consent was obtained from the parents, and all individual information was processed with strict confidentiality. The study was approved by the Icelandic Bioethics Committee and Ethical Committee at Landspitali University Hospital and registered at the Icelandic Data Protection Authority.

### 2.2. Vitamin D Measurement

Fasting blood samples were collected when the children were 1 year old (January–December 2006) and 6 years old (June–December 2011). Serum samples were stored at −80 °C until analyzed for 25(OH)D with an electro-chemiluminescence immunoassay (Modular Analytics E170, Roche Diagnostis, Mannheim, Germany) (precision 0.1 nmol/L). The main cut-point explored and used to describe vitamin D sufficiency was ≥50.0 nmol/L. Other cut-points were ≥75.0 nmol/L, 30.0−49.9 nmol/L and <30.0 nmol/L [8,27]. To classify the children according to expected contribution of cutaneous synthesis to vitamin D status at 6 years, three categories were constructed; blood samples collected in July−August (major contribution; “summer”), September−October (moderate contribution; “autumn”), and November−December and June (minor contribution; “winter/spring”).

### 2.3. Covariates

Potential confounders were gender, breastfeeding, vitamin D intake and supplement use, serum levels of triglycerides (TG), total cholesterol (TC), low-density lipoprotein-cholesterol (LDL-C), high-density lipoprotein-cholesterol (HDL-C), body mass index (BMI), body weight, physical activity assessed at 6 years, and maternal age, maternal BMI and maternal education, reported when the child was 6 years [5,28,29,30]. Information on breastfeeding duration and exclusivity was gathered monthly in the first year of life. Dietary data were obtained from 3-day weighed food records (HR 2385 scales, Philips, Hungary or Austria) (precision 1 g) registered at 1 and 6 years of age. Total vitamin D intake included vitamin D from food and supplements. Serum TG and TC were analyzed using an enzymatic colorimetric test (Cholesterol CHOD-PAP, Roche Diagnostics, Mannheim, Germany) (precision 0.1 nmol/L). Serum HDL-C was analyzed using the same method after precipitation and centrifugation. Serum LDL-C was calculated using the Friedewald formula [31]. BMI was calculated using height and weight measured with a Marel M series 1100 scale (Reykjavik, Iceland) (precision 0.1 kg) and an Ulmer stadiometer (Prof. Heinze, Ulm, Germany) (precision 0.5 cm). Information on children’s daily duration of physical activity, maternal age, maternal BMI and maternal education was obtained from parent questionnaires. Maternal education was defined as the highest completed level and categorized as primary (10 years), secondary (approx. 14 years) and tertiary (>16 years) education.

### 2.4. Statistical Analysis

Statistical analysis was performed with SAS (Enterprise Guide 4.3; SAS Institute Inc, Cary, NC, USA). Variables were examined for normality using Quantile Quantile-plots and described using mean and standard deviations (SD) or medians and 25th to 75th centiles. For comparison between groups, we used the *t*-test, Mann Whitney U-test or a chi-square. Missing values were replaced by the median value for each covariate (1%–4%) except for vitamin D supplement use and maternal education. Multivariate logistic regression was used to assess variables associated with vitamin D sufficiency (25(OH)D ≥ 50.0 nmol/L) at 6 years. Model 1 included gender and variables that were found to be significantly different between vitamin D sufficient children and children with lower status. In model 2 we examined the importance of including 25(OH)D at 1 year. Further adjustments for other covariates (serum TG, TC, LDL-C, HDL-C, BMI, body weight, physical activity, or maternal age, maternal BMI, or maternal education) did not contribute significantly to the models. The mean change in vitamin D intake and status from 1 to 6 years of age was calculated as the difference between the values at 6 years and 1 year. Correlations for vitamin D intake and status from 1 to 6 years were evaluated with Spearman and Pearson correlations, respectively. When categorizing subjects into tertiles based on serum 25(OH)D levels, the cut-off values of ≤81.3 nmol/L and ≤119.1 nmol/L at 1 year and ≤47.3 nmol/L and ≤63.2 nmol/L at 6 years were used. Linear regression models were used to test for predictors of change in vitamin D status from 1 to 6 years. Two sided *p*-value of <0.05 was considered statistically significant.

## 3. Results

At 6 years, 89 children (64%) were classified as vitamin D sufficient (25(OH)D ≥ 50.0 nmol/L). From these, 17 children (12%) had levels ≥75.0 nmol/L. Fourty-two children (30%) had 25(OH)D levels 30.0-49.9 nmol/L and 8 children (6%) had levels <30.0 nmol/L. Children using vitamin D supplements, cod liver oil being most common, received a median of 11.9 μg/day vitamin D (25th, 75th percentiles: 7.4, 15.9), thereof 8.8 μg/day (25th, 75th percentiles: 5.3, 13.3) from supplements. Vitamin D intake from food did not differ between children using and not using vitamin D supplements. The main food sources for vitamin D were breakfast cereals (median 0.8 μg/day; 25th, 75th percentiles: 0.4, 1.6), butter (median 0.3 μg/day; 25th, 75th percentiles: 0.1, 0.5), fish (median 0.2 μg/day; 25th, 75th percentiles: 0.0, 0.4), meat (median 0.1 μg/day; 25th, 75th percentiles: 0.1, 0.2) and milk (median 0.1 μg/day; 25th, 75th percentiles: 0.1, 0.1). No dietary vitamin D sources apart from supplements were associated with vitamin D status. Table 1 presents the characteristics of eligible subjects. Vitamin D sufficient 6 year old children had been breastfed longer, were more likely to follow recommendations on vitamin D supplement use and therefore had higher total vitamin D intake than children with lower vitamin D status. Their blood samples were more likely to have been obtained during summer and less likely to have been obtained during winter or spring. From those children following vitamin D supplement recommendations (10 μg/day) were 83% vitamin D sufficient, with no differences in status between season of blood sampling (*p* > 0.05). Season was an important predictor of vitamin D sufficiency among children not receiving the recommended 10 µg/day of vitamin D. Among those children, 25% having blood drawn during winter or spring were vitamin D sufficient, compared with 52% of children measured during autumn and 76% of children measured during summer (*p* < 0.001).

**Table 1:** Characteristics of eligible subjects.

Variable	All Subjects (*n* = 139) ^1^	25(OH)D < 50 nmol/L (*n* = 50)	25(OH)D ≥ 50 nmol/L (*n* = 89)	*p*-Value
Male gender, *n* (%)	70 (50)	22 (31)	48 (69)	0.26 ^2^
Female gender, *n* (%)	69 (50)	28 (41)	41 (59)	0.26 ^2^
Breastfeeding, month, median (25th, 75th centile)	8 (6, 10)	7 (4, 9)	9 (7, 10)	0.017 ^3^
Exclusive breastfeeding, month, median (25th, 75th centile)	4 (2, 5)	4 (2, 5)	4 (3, 5)	0.14 ^3^
**Children at 6 years**				
Age, year, mean ± SD	6.1 ± 0.3	6.1 (0.2)	6.2 (0.3)	0.60 ^4^
BMI, kg/m^2^, median (25th, 75th centile)	15.5 (14.8, 16.5)	15.5 (15.0, 16.8)	15.5 (14.7, 16.4)	0.49 ^3^
Physical activity, h, median (25th, 75th centile)	1.6 (1.0, 2.5)	1.6 (1.0, 2.0)	1.6 (1.1, 2.7)	0.16 ^3^
Total vitamin D intake, µg/day, median (25th, 75th centile)	5.0 (2.3, 12.1)	3.2 (2.1, 6.6)	7.5 (2.7, 13.7)	0.003 ^3^
Vitamin D from food, µg/day, median (25th, 75th centile)	2.3 (1.6, 3.3)	2.4 (1.7, 3.0)	2.3 (1.6, 3.4)	0.69 ^3^
Vitamin D from supplements, µg/day, median (25th, 75th centile)	1.5 (0.0, 10.0)	0.0 (0.0, 4.0)	4.7 (0.0, 10.0)	0.002 ^3^
No vitamin D supplement use, *n* (%)	63 (47)	31 (49)	32 (51)	0.003 ^2^
Vitamin D supplement use < 10 µg/day, *n* (%)	37 (27)	11 (30)	26 (70)	0.36 ^2^
Vitamin D supplement use ≥ 10 µg/day, *n* (%)	35 (26)	6 (17)	29 (83)	0.007 ^2^
Blood samples in winter/spring, *n* (%)	38 (27)	21 (55)	17 (45)	0.004 ^2^
Blood samples in autumn, *n* (%)	34 (25)	13 (38)	21 (62)	0.75 ^2^
Blood samples in summer, *n* (%)	67 (48)	16 (24)	51 (76)	0.004 ^2^
Serum 25(OH)D, nmol/L, mean ± SD	56.5 ± 17.9	39.3 ± 9.5	66.1 ± 13.8	<0.001 ^4^
Serum TG, mmol/L, mean ± SD	0.6 ± 0.2	0.7 ± 0.3	0.6 ± 0.2	0.26 ^4^
Serum TC, mmol/L, mean ± SD	4.4 ± 0.6	4.4 ± 0.6	4.4 ± 0.7	0.57 ^4^
Serum LDL-C, mmol/L, mean ± SD	2.5 ± 0.6	2.6 ± 0.5	2.5 ± 0.6	0.47 ^4^
Serum HDL-C, mmol/L, mean ± SD	1.6 ± 0.3	1.6 ± 0.3	1.6 ± 0.3	0.80 ^4^
**Mothers of 6 year old children**				
Age, year, mean ± SD	36.6 ± 5.0	36.2 ± 5.4	36.8 ± 4.8	0.50 ^4^
BMI, kg/m^2^, median (25th, 75th centile)	24.4 (21.8, 27.8)	24.8 (23.3, 28.1)	24.4 (21.5, 27.3)	0.16 ^3^
Primary education, *n* (%)	18 (13)	9 (50)	9 (50)	0.18 ^2^
Secondary education, *n* (%)	31 (23)	11 (35)	20 (65)	0.95 ^2^
Tertiary education, *n* (%)	87 (64)	29 (33)	58 (67)	0.40 ^2^

Abbreviations: 25(OH)D—25-hydroxyvitamin D; BMI—body mass index; HDL-C—high-density lipoprotein-cholesterol; LDL-C—low-density lipoprotein-cholesterol; TC—total cholesterol; TG—triglycerides; ^1^ For vitamin D supplement use and maternal education: *n* = 135 and *n* = 136, respectively; ^2^ Chi-square used to determine the *p*-value; ^3^ The Mann Whitney U-test used to determine the *p*-value; ^4^ The *t*-test used to determine the *p*-value.

Multivariate logistic regression models (Table 2) confirmed that vitamin D intake at 6 years (intake range: 1 to 26 μg/day) and seasons were both independent predictors of vitamin D sufficiency at 6 years. As an example, increasing total vitamin D intake by 10 μg/day in model 1 was associated with around 4-fold higher odds of having serum 25(OH)D above 50 nmol/L. This estimate was comparable to what was seen to seasonal differences. Vitamin D status at 1 year was also found to be an independent predictor of vitamin D sufficiency at 6 years (model 2). Including the vitamin D status at 1 year in the model strengthened the estimates for vitamin D intake and seasons.

**Table 2:** Multivariate logistic regression analysis of factors associated with vitamin D sufficiency (serum 25(OH)D ≥ 50 nmol/L) at 6 years of age.

	Model 1 (*n* = 139)		Model 2 (*n* = 74)	
Variable	OR	95% CI	OR	95% CI
Female gender	0.68	0.31–1.48	0.59	0.18–1.94
Breastfeeding, month	1.07	0.95–1.22	1.10	0.91–1.34
Total vitamin D intake, 10 µg/day	3.85	1.73–8.58	10.93	2.21–53.99
Blood samples in winter/spring	reference		reference	
Blood samples in autumn	3.03	1.04–8.84	5.64	1.16–27.32
Blood samples in summer	5.84	2.12–16.07	8.88	1.83–43.23
Serum 25(OH)D at 1 year, nmol/L			1.02	1.002–1.04

Abbreviations: 25(OH)D—25-hydroxyvitamin D; CI—confidence interval; OR—Odds Ratio; Model 2: Same as model 1 as well as serum 25(OH)D at 1 year as independent variable.

**Figure 1:**
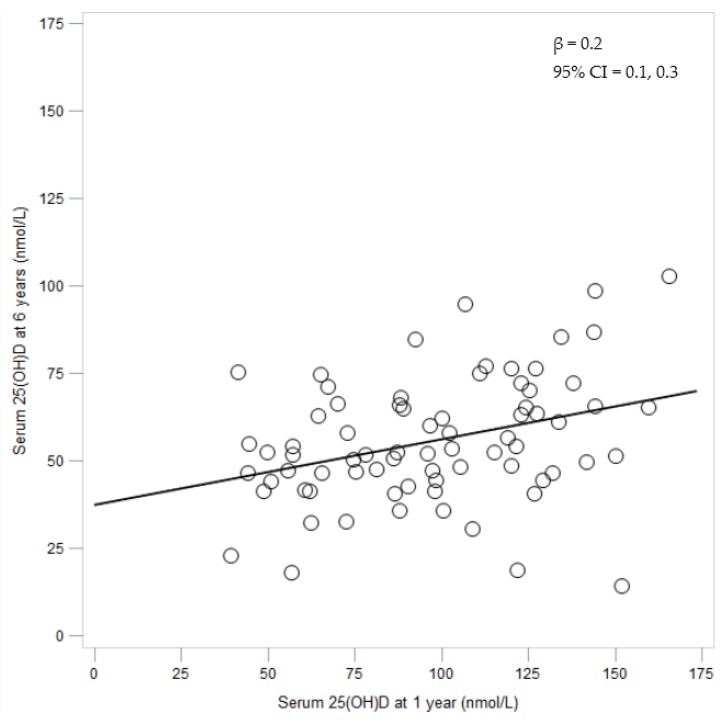
Relationship between the serum 25(OH)D levels assessed at 1 year and 6 years (*n* = 74).

The basic characteristics of the 74 subjects with vitamin D status measured both at 1 and 6 years did not differ from other eligible subjects (see Table 1). As presented in Table 3, vitamin D intake and status measured as 25(OH)D dropped from 1 to 6 years. Vitamin D intake at 1 and 6 years did not correlate, neither when explored as µg/day nor µg/kg/day, while the correlation for serum 25(OH)D levels at 1 and 6 years was 0.34 (*p* = 0.003). The relationship between the serum 25(OH)D levels assessed at 1 year and 6 years is shown in Figure 1.

**Table 3:** Vitamin D intake and status of children with vitamin D status measured at two time points (*n* = 74).

Variable	Age 1 Year	Age 6 Years	Change from 1 to 6 Years
Total vitamin D intake µg/day, median (25th, 75th centile)	6.4 (3.5, 11.6)	4.5 (2.1, 11.0)	−0.9 (−8.1, 4.4)
Total vitamin D intake, µg/kg/day, median (25th, 75th centile)	0.7 (0.3, 1.2)	0.2 (0.1, 0.5)	−0.3 (−1.0, 0.0)
Serum 25(OH)D, nmol/L, mean ± SD	97.5 ± 32.4	55.8 ± 17.9	−41.6 ± 31.2

Abbreviations: 25(OH)D—25-hydroxyvitamin D.

As shown in Table 4, 40%–54% of subjects stayed within the same vitamin D tertile at 1 and 6 years. Only 16%–21% moved from the lowest or highest tertile to the other extreme. The four children moving from tertile 1 at 1 year to tertile 3 at 6 years all had an increase in total vitamin D intake from 1 to 6 years (range 1.2 to 4.7 μg/day and 0.0 to 0.1 μg/kg/day). The five children moving from tertile 3 at 1 year to tertile 1 at 6 years all had a decrease in total vitamin D intake from 1 to 6 years (range −27.4 to −1.8 μg/day and −2.1 to −0.3 μg/kg/day).

**Table 4:** Numbers of children with vitamin D status measured at two time points falling in different vitamin D tertiles at 1 and 6 years of age (*n* = 74).

	Serum 25(OH)D Tertile at 6 Years		
Serum 25(OH)D tertile at 1 year	1, *n* (%)	2, *n* (%)	3, *n* (%)
1, *n* (%)	12 (48)	9 (36)	4 (16)
2, *n* (%)	8 (32)	10 (40)	7 (28)
3, *n* (%)	5 (21)	6 (25)	13 (54)

Abbreviations: 25(OH)D—25-hydroxyvitamin D.

There was a strong negative association between the serum 25(OH)D level assessed at 1 year and the change in serum 25(OH)D levels from 1 to 6 years of age (β = −0.8, 95% CI = −0.9, −0.7). The only other baseline variable associated with 25(OH)D change over time in a univariate linear model was vitamin D intake at 1 year (β = −1.9, 95% CI = −3.2, −0.6 when explored as µg/day and β = −19.1, 95% CI = −32.4, −5.7 when explored as µg/kg/day). The change in vitamin D intake from 1 to 6 years of age was also associated with the change in serum 25(OH)D levels from 1 to 6 years of age (β = 1.3, 95% CI = 0.4, 2.2 when explored as µg/day and β = 18.6, 95% CI = 6.5, 30.7 when explored as µg/kg/day). Gender, breastfeeding, anthropometrics, or maternal factors were not associated with 25(OH)D change over time.

## 4. Discussion

Our main finding was the tracking of vitamin D status during a 5-year period from 1 to 6 years of age, with a clear regression towards the mean. A Norwegian study on adults (mean age at baseline 57 years) reported tracking of vitamin D status during a 14-year period [32] but to the best of our knowledge this is the first study to present tracking of vitamin D status from infancy to childhood. Tracking is used in public health and epidemiology to describe the stability of a variable over time, attempting early identification of subjects at risk for diseases later in life [33]. The correlation we observed in serum 25(OH)D levels from 1 to 6 years of age was similar in strength as that observed for other biochemical variables in children, e.g., serum lipid and apolipoprotein levels from infancy to 4 years of age in a Swedish study [34]. We believe that our results merely provide motivation for further studies on tracking of vitamin D status in infancy and childhood and its clinical significance. They may provide support for the importance of monitoring vitamin D status.

Serum 25(OH)D dropped from high levels at 1 year (mean 97.5 nmol/L) to lower levels at 6 years (mean 56.5 nmol/L). At 1 year, 92% of the infants were vitamin D sufficient (≥50.0 nmol/L) and 24% had levels that may be considered adversely high (>125 nmol/L) [17] while at 6 years only 64% of the children were vitamin D sufficient (≥50.0 nmol/L). This is in line with cross-sectional epidemiological studies reporting higher 25(OH)D levels in younger children than in older children and adolescents [22,23,24]. This might call into question using the same cut-off value to describe vitamin D sufficiency in infants as in older children. It should also be kept in mind that while 50 nmol/L (20 ng/mL) is frequently used as indicative of vitamin D sufficiency [8,35,36] higher cut-off values, e.g., 75 nmol/L (30 ng/mL) have been suggested for endpoints other than bone health [27].

Our study found that the higher the serum 25(OH)D level was at 1 year, the more it changed (decreased) over the 5-year period to 6 years of age, lessening our concerns about possible adverse effects due to high levels observed in infancy. A Polish infant study reported similar findings in the age period 6 to 12 months [37]. The explanations for our findings may be complex. Firstly, the decrease in 25(OH)D levels may be explained by a substantial decrease in vitamin D intake from 1 to 6 years, especially when measured in µg per kg body weight. Although age and body weight have been shown to be important predictors of variation in 25(OH)D levels [38,39], current vitamin D recommendations do not take this into account [8,13]. Secondly, it has been suggested that high serum 25(OH)D levels at an earlier point may stimulate a negative feedback, resulting in lower levels at a later point [37]. Thirdly, there is a possibility that serum 25(OH)D levels in part of the children at 1 year were overestimated, *i.e.,* due to the possible presence of C-3 epimers [40,41,42]. Other studies have shown higher concentrations of C-3 epimers of 25(OH)D in infants under 1 year of age than older children and adults [41,42]. However, we do not know whether, or to what extent, these were present in our subjects.

As presented in our previous publication, vitamin D intake, but not breastfeeding or season, was associated with vitamin D status at 1 year [17]. In the current study we found that vitamin D status at 1 year and vitamin D intake at 6 years were associated with vitamin D status at 6 years. Season was only associated with vitamin D status among children not receiving the recommended intake of vitamin D. In our study population increasing vitamin D intake by 10 μg/day (amount corresponding to the daily recommended intake) and seasonal differences had similar influence on the odds of having serum 25(OH)D above 50 nmol/L. Vitamin D intake has been positively associated with serum 25(OH)D levels in international systematic reviews and meta-analyses [43,44], supporting a public health emphasis on vitamin D intake in regions and among groups of people where sufficient cutaneous synthesis of vitamin D is not guaranteed. This is the case in Iceland. Few foods are naturally rich in vitamin D [45] and consumption of vitamin D fortified foods and drinks, such as milk, dairy products or orange juice was not common in Iceland at the time of the study. Therefore vitamin D supplements are important vitamin D sources in Iceland and are included in the food-based dietary guidelines for children and adults [16]. As in our study, season has clearly been shown to affect vitamin D status in our neighboring countries [23,29,46]. Consequently, official guidelines emphasize vitamin D supplement use during winter but less so during summer [16]. However, mean serum 25(OH)D levels in June (spring) were lowest of all months with vitamin D status measured (45.3 nmol/L) and still only three-quarters of children were vitamin D sufficient in July–August (summer). Therefore, to ensure sufficient vitamin D status all year round it might be important to use vitamin D supplements throughout the year in Iceland. Both genetic and socio-demographic factors have been associated with vitamin D status in other studies [29,30,47,48]. Our finding of longer breastfeeding duration in the first year of life among vitamin D sufficient 6 year olds possibly reflects long-term effects of socio-demographic factors on vitamin D status. Although maternal education was not associated with vitamin D sufficiency in the current analysis, breastfeeding duration is associated with maternal education in this cohort, as previously presented [49].

The main strength of the study is that vitamin D status was measured at two different times in the subjects, giving tracking data from 1 to 6 years of age. Another strength is the detailed information on the cohort, *i.e.,* infant and socio-demographic data, and simultaneous analysis of vitamin D intake and status. We have previously reported that the original sample is population based and therefore representative of Icelandic children [25,50]. However, as the current analysis on vitamin D status at 6 years is a secondary analysis in the original study, the interpretations of the results are subject to some limitations. Vitamin D status was not measured all months of the year among the 6 year old children. This may possibly affect the observed magnitude of tracking. Data on vitamin D intake from food on the individual level may be subject to underreporting as intake of fish, especially fatty fish, may be hard to capture with three diet registration days [51]. Frequent fish consumption in Iceland as well as the likelihood of a lower day to day variance in the diet of young children compared to adults may diminish this potential problem. The parent questionnaire was not developed to assess cutaneous vitamin D synthesis and our best available indicator on sun exposure may be the reported physical activity. Information about time spent outdoors, weather conditions, skin pigmentation and habitual clothing and sunscreen use would have been a valuable addition [52].

In conclusion, vitamin D status in infancy as well as current vitamin D intake and season are clear predictors of vitamin D status in early school age Icelandic children. To our best knowledge this is the first study to present tracking of vitamin D status from infancy to childhood, providing motivation for further studies on tracking and its clinical significance.
